# CpG Site-Specific Regulation of Metallothionein-1 Gene Expression

**DOI:** 10.3390/ijms21175946

**Published:** 2020-08-19

**Authors:** Shoko Ogushi, Yuya Yoshida, Tsuyoshi Nakanishi, Tomoki Kimura

**Affiliations:** 1 Department of Life Science, Faculty of Science and Engineering, Setsunan University, Neyagawa 572-8508, Japan; ogushi.shouko@ma.medience.co.jp; 2 Department of Pathological Biochemistry, Faculty of Pharmaceutical Sciences, Setsunan University, Hirakata 573-0101, Japan; yoshida@pharm.setsunan.ac.jp; 3 Laboratory of Hygienic Chemistry and Molecular Toxicology, Gifu Pharmaceutical University, Gifu 501-1196, Japan; nakanishi@gifu-pu.ac.jp

**Keywords:** metallothionein, epigenetics, heavy-metal toxicity

## Abstract

Metal-binding inducible proteins called metallothioneins (MTs) protect cells from heavy-metal toxicity. Their transcription is regulated by metal response element (MRE)-binding transcription factor-1 (MTF1), which is strongly recruited to MREs in the MT promoters, in response to Zn and Cd. Mouse *Mt1* gene promoter contains 5 MREs (a–e), and MTF1 has the highest affinity to MREd. Epigenetic changes like DNA methylation might affect transcription and, therefore, the cytoprotective function of MT genes. To reveal the CpG site(s) critical for *Mt1* transcription, we analyzed the methylation status of CpG dinucleotides in the *Mt1* gene promoter through bisulfite sequencing in P1798 mouse lymphosarcoma cells, with high or low MT expression. We found demethylated CpG sites near MREd and MREe, in cells with high expression. Next, we compared *Mt1* gene-promoter-driven Lucia luciferase gene expression in unmethylated and methylated reporter vectors. To clarify the effect of complete and partial CpG methylation, we used M.SssI (CG→^5m^CG) and HhaI (GCGC→G^5m^CGC)-methylated reporter vectors. Point mutation analysis revealed that methylation of a CpG site near MREd and MREe strongly inhibited *Mt1* gene expression. Our results suggest that the methylation status of this site is important for the regulation of *Mt1* gene expression.

## 1. Introduction

Metallothioneins (MTs) are low-molecular-weight, cysteine-rich, metal-binding proteins [1]. In mammals, there are four main isoforms, MT1 to MT4. The expression of ubiquitous MT1 and MT2 isoforms is induced by heavy metals. These isoforms protect against toxic heavy metals by sequestering them and lowering their concentrations at critical intracellular sites [2,3,4]; they also provide protection against reactive oxygen species (ROS) [5,6,7]. The roles of MT1/2 in immunity and obesity were reported using MT1/2 knock-out mice [8], and changes in the MT expression levels might affect the biological functions of MTs.

Epigenetics refers to the control of gene expression via mechanisms not directly related to the DNA coding sequence [9]. Epigenetic modifications regulate gene expression through various epigenomic marks; for example, methylation of cytosine residues at CpG sites with histone modifications, to regulate gene expression [10,11]. The methylated CpG sites recruit methyl CpG-binding domain proteins and thereby prevent the binding of transcription factors to the DNA [12]. CpG methylation or demethylation in the MT promoters might cause individual differences in MT expression levels, and therefore differences in susceptibility to heavy metal toxicity.

Metal response element (MRE)-binding transcription factor-1 (MTF1) activates MT1/2 transcription in response to heavy metals [13]. Among 5 MREs (a–e) in the 5′-flanking region of the mouse *Mt1* gene, MTF1 shows the highest affinity to MREd [14,15]. MTF1 is an essential transcription factor for basal and metal-induced MT expression [16]. A transcriptional/epigenetic regulator p300, histone acetyltransferase (HAT), is required for MT induction in response to metals [17]. The importance of the formation of the complex containing p300 was shown, such that hexavalent chromium (Cr^6+^) inhibited MT induction via inhibition of this complex formation [18,19]. However, the inhibitory effects of Cr^6+^ on MT induction were due to its effects on the HAT-independent transactivation ability of p300. The importance of p300 histone acetyltransferase activity in MT expression remains unclear. MT expression is modulated by epigenetic modifications [20,21,22,23,24]. Zinc deficiency enhances the cadmium-induced expression of mouse hepatic MT2, without change of basal MT2 expression in vivo; it leads to hypermethylation of CpG sites and enrichment in the acetylation of histones H3/H4 in the 5′-flanking region of the *Mt2* gene [25]. We reported that the *Mt1* gene is suppressed in P1798 mouse lymphosarcoma cells, but long-term cadmium (Cd) exposure (0.1 μM, 7days) and subsequent short-term exposure to high Cd concentrations (10 μM, 3 hr) decreases MT1 DNA methylation and induces the *Mt1* gene [26]. We hypothesized that certain CpG sites in the MT1 promoter were crucial for *Mt1* gene expression, because the CpG sites were reported to be crucial for the regulation of several genes [27,28]. However, the CpG site(s) whose methylation status is important for MT1 expression is not yet determined.

Here, we found that the methylation status of CpG dinucleotides in the 5′-flanking region of the *Mt1* gene differed in P1798 cells expressing high and low levels of MT1. Luciferase analysis revealed that the effect of CpG methylation was not uniform. We found that methylation status of a CpG site near MREd and MREe is important for the regulation of *Mt1* gene expression.

## 2. Results and Discussion

### 2.1. Characterization of P1798 Cells Treated with 5-AzaC Plus Cd

**Figure 1:**
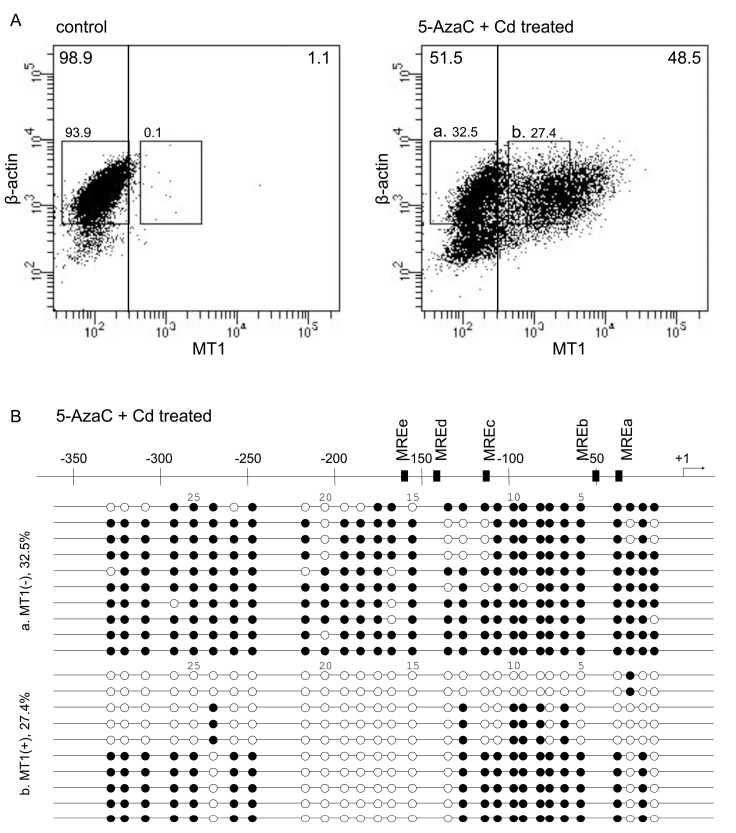
PrimeFlow RNA assay of 5-AzaC and Cd-treated P1798 cells and bisulfite sequencing. (**A**) P1798 cells were treated with vehicle (control) or 5-AzaC (2.5 μM, 2 days) and Cd (10 μM, 3 h). Experiments were reproduced three times with similar results. Results from a representative experiment are shown. (**B**) Results of bisulfite sequencing of (a) and (b) cell fractions. Designations of CpG dinucleotides are represented by circles: closed—methylated; open—unmethylated. Each row represents data from a single cell.

In P1798 cells, MT1 expression is extremely low but is increased by 5-azacytidine (5-AzaC) plus Cd (5-AzaC/Cd) treatment, because of CpG demethylation by 5-AzaC and MTF1 activation by Cd [22]. The level of MT1 mRNA was increased in 5-AzaC/Cd-treated cells (Table 1). Untreated P1798 cells showed extremely low MT1 expression—98.9% cells had MT1 expression (arbitrary units) under 3 × 10^2^ (Figure 1A, left panel). After 5-AzaC/Cd treatment, 51.5% of the cells still had low MT1 expression and 48.5% of the cells had high MT1 expression (over 3 × 10^2^ on the MT1 axis). Bisulfite sequencing revealed that cytosines at CpG sites in some regions of the MT1 promoter tended to be methylated in cells with low levels of MT1 expression but unmethylated in cells with high levels. The first CpG site and the 14th to 21st CpG sites (between −215 to −127 bp) from the transcription start site were unmethylated in all cells with high MT1 expression (Figure 1B). Similar ununiformed demethylation after 5-AzaC treatment in P1798 cells was reported by Ghoshal et al. [22]. They analyzed methylation status on the 1st to 18th CpG sites and reported that 5-AzaC treatment for 120 hr showed complete demethylation in the region. Thirty-six hour treatment showed partial demethylation only in 1st, 3rd–5th, 7th, and 13th–18th CpG sites. The latter region was near MREd and MREe. As MREd is the most active element in the response to Zn [14,15], we hypothesized that the demethylation of these CpG sites are integrally involved in the expression of MT1.

### 2.2. Effect of CpG Methylation in the 5′-Flanking Region of the MT1 Gene on Lucia Luciferase Reporter Gene Expression

**Figure 2:**
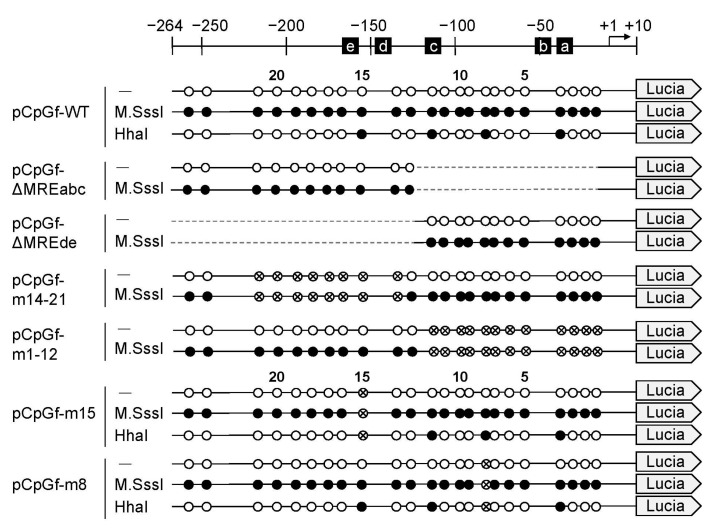
Structure of the MT1 promoter region and the introduced deletions and point mutations. Schematic at the top shows the transcription start site (arrow) and metal response element (MREs). CpG dinucleotides are as in Figure 1; crossed, mutated. Lucia, Lucia luciferase gene.

**Figure 3:**
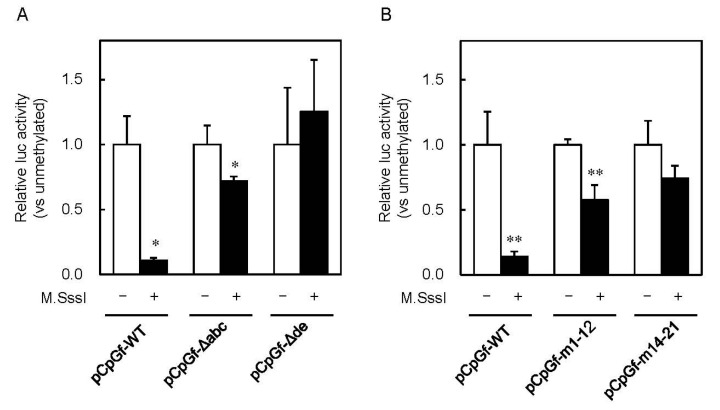
Effect of CpG methylation in MT1 promoter on Lucia luciferase activity. Mouse embryonic fibroblasts (MEFs) were transfected with unmethylated or M.SssI-methylated Lucia reporter vectors carrying (**A**) deletions or (**B**) point mutations. Luciferase reporter vector (pGL4.12-SV40) was co-transfected for normalization of transfection efficiency. Reporter gene expression was measured using the Dual-Luciferase Reporter Assay System, and Lucia (luc) activity was normalized to firefly luciferase activity. Values are expressed as the means ± SD from three independent experiments. * *p* < 0.05, ** *p* < 0.01 versus the unmethylated vector group.

Next, we compared the *Mt1* gene promoter-driven Lucia luciferase gene expression in unmethylated and methylated reporter vectors in mouse embryonic fibroblasts (MEFs) (Figure 2). CpG methylation by M.SssI inhibited MT1 promoter-mediated expression of Lucia luciferase by 89% vs. the unmethylated group (Figure 3A). Non-uniform regulatory effects of the methylation status of CpG sites were reported [27,28]. Methylation of some CpG sites remarkably down-regulated gene expression, whereas methylation of other CpG sites had no effect in the *NOS2* and *MMP9* gene. Our report showed that Cd increased MT inducibility with demethylation of the −200 to −55 bp region of the MT1 promoter [26]. However, we did not identify specific CpG sites involved in MT1 expression. To clarify which CpG site is important, we used reporter vectors with deletions or point mutations. In pCpGf-ΔMREde, the inhibitory effect of M.SssI was not observed, but 28% of inhibition remained in pCpGf-ΔMREabc (Figure 3A). Similar results were observed in mutated reporter vectors. The inhibitory effect of M.SssI was not significant in pCpGf-m14-21 (CpG sites near MREd and e mutated; 26% inhibition), but was significant in pCpGf-m1-12 (CpG sites near MREa, b, and c mutated; 42% inhibition; Figure 3B).

**Figure 4:**
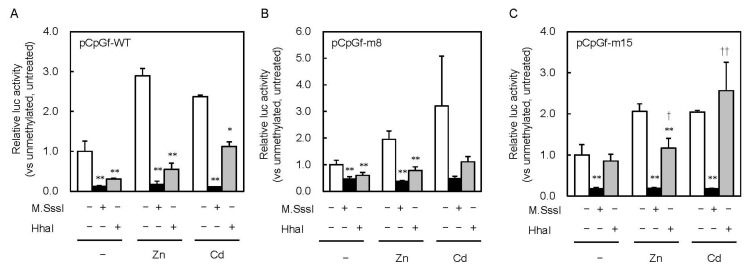
Effect of partial CpG methylation in the MT1 promoter on Zn- and Cd-induced Lucia luciferase activity. MEFs were transfected with unmethylated or M.SssI- or HhaI methyltransferase–methylated Lucia reporter vectors carrying wild-type (**A**), point mutations of CpG8 (**B**) and CpG15 (**C**). Reporter gene expression was measured 6 h after Zn or Cd addition. Values are expressed as the means ± SD from three independent experiments. * *p* < 0.05, ** *p* < 0.01 versus the unmethylated group. ^†^ *p* < 0.05, ^††^ *p* < 0.01 versus the M.SssI-methylated group.

To clarify the effect of partial CpG methylation on MT1 promoter activity, we methylated the reporter vectors with HhaI methyltransferase (Figure 2). M.SssI inhibited the promoter activity by 88% and HhaI inhibited it by 69%. Similar inhibitory effects were observed in cells treated with 200 μM Zn or 10 μM Cd. The inhibitory effect of M.SssI was still observed with pCpGf-m15 (Figure 4C). In pCpGf-m8, the inhibitory effect of M.SssI was not significant in the Cd-treated group, but its inhibitory tendency was observed (Figure 4B). The non-uniform effect of CpG methylation was much clearer with HhaI. The inhibitory effect of HhaI on pCpGf-WT and pCpGf-m8 vectors was similar to that of M.SssI in the untreated and Zn-treated groups (Figure 4B), but not on pCpGf-m15 (Figure 4C). The inhibitory effect of HhaI on pCpGf-m15 was not observed in the untreated and Cd-treated groups (Figure 4C). These results suggest that the 15th CpG site (located between MREd and MREe) greatly affected MT1 expression. This location, CpG15, did not exactly match the specific demethylation site in our previous work in long-term Cd-treated P1798 cells [26]. Methylation/demethylation near CpG15, not CpG15, might be important for MT1 expression. Site-specific methylation/demethylation techniques [29,30] are needed to answer this question.

### 2.3. Involvement of the MTF1–MRE Pathway on the Inhibition of MT1 Expression by CpG Methylation

**Figure 5:**
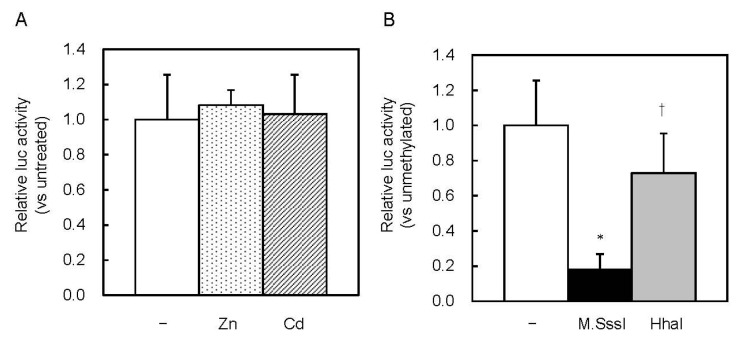
Effect of CpG methylation on MT1 promoter activity in MTF1 KO cells. (**A**) Cells were transfected with unmethylated Lucia reporter vectors. Reporter gene expression was measured 6 h after Zn or Cd addition. Values for neither treatment group were significantly different from the untreated group. (**B**) MTF1-KO cells were transfected with unmethylated or M.SssI- or HhaI methyltransferase-methylated Lucia reporter vectors. Values are expressed as the means ± SD from three independent experiments. * *p* < 0.01 versus the unmethylated group. ^†^ *p* < 0.01 versus the M.SssI-methylated group.

MREd is the most active element in response to Zn [14,15]. MT1 promoter activity might be inhibited by HhaI methyltransferase, through inhibition of the MTF1–MRE pathway. We investigated whether this pathway was involved in the inhibition caused by CpG methylation. Basal MT expression was low in MTF1-knockout (MTF1-KO) cells, and Zn or Cd treatments did not increase it [16]. In MTF1-KO cells, the Zn and Cd treatments did not increase MT1 promoter-driven Lucia luciferase activity (Figure 5A). CpG methylation by M.SssI inhibited the activity, whereas methylation by HhaI did not (Figure 5B). Complete CpG methylation by M.SssI might inhibit recruitment of basic transcription apparatus or change the chromatin structure.

## 3. Materials and Methods

### 3.1. Cell Culture and Treatment

P1798 mouse lymphosarcoma cells—a gift from Dr. A.E. Thompson, University of Texas (Galveston, TX, USA)—were cultured as in [26]. P1798 cells were seeded at a density of 2.5 × 10^5^ cells/mL every 2 days. P1798 cells were treated with 5-AzaC (2.5 μM, 2 days) and subsequent exposure to Cd (10 μM, 3 h). MEFs and MTF1-KO MEFs were provided by Dr. G.K. Andrews, University of Kansas Medical Center (Kansas City, KS, USA); these were cultured as in [17]. MEFs were seeded at a density of 4.0 × 10^4^ cells/well of 24 well plate. Cell viability was measured with trypan blue exclusion assays [31]. Before the transfection for the luciferase reporter assay, the cells were cultured for 24 h.

### 3.2. Quantitative Reverse Transcription PCR

RNA was isolated from cells by using Isogen RNA extraction reagent (Nippon Gene, Tokyo, Japan), in accordance with the manufacturer’s protocol. Extracted total RNA was converted to the complementary DNA, by using a High Capacity cDNA Reverse Transcription Kit (Thermo Fisher Scientific, Waltham, MA, USA) and a random primer. For quantitative PCR (qPCR), we used Premix Ex Taq (Probe qPCR) (Takara Bio, Kusatsu, Japan) or TB Green Premix Ex Taq (Tli RNaseH Plus) (Takara Bio) as the qPCR reagent and an Eco Real-Time PCR System (Illumina, San Diego, CA, USA). For measurement of MT1 cDNA, we used TaqMan probes (for MT1: Mm00496660_g1; for 18S rRNA: 4319413E; Applied Biosystems, Carlsbad, CA, USA). Gene-specific primers for β-actin was as follows—forward (F) primer, 5′-GAAATCGTGCGTGACATCAAAG-3′; reverse (R) primer, 5′-TGTAGTTTCATGGATGCCACA G-3′.

### 3.3. PrimeFlow RNA Assay

Probes and reagents were part of the PrimeFlow RNA Assay Kit (eBioscience, San Diego, CA, USA) [32]. P1798 cells (5.0 × 10^6^) were incubated with permeabilization buffer containing RNase inhibitors, fixed and hybridized with target gene-specific probes. Cells were stained with PreAmp Mix, Amp Mix, and probes labeled with Alexa Fluor 647 (Type 1) for MT1 and Alexa Fluor 488 (Type 4) for β-actin, according to the manufacturer’s protocol. Flow cytometric analysis was performed using a BD FACSAria Fusion cell sorter (BD Biosciences, San Jose, CA, USA). Cells were sorted and collected according to the MT1 expression level.

### 3.4. Bisulfite Genomic Sequencing Assay

Bisulfite conversion reactions, PCR amplification, cloning of the PCR product and sequencing were performed as in [26]. The sequences of the sense and antisense primers were as follows: F primer, 5ʹ-TTAGGAATTTTAGGAAAGGAGA-3ʹ; R primer, 5ʹ-TAAAAAACAACCTACCCTCTTT-3ʹ. The QUMA program (http://quma.cdb.riken.jp/) was used to align, visualize, and quantify sequence data for CpG methylation analysis [33].

### 3.5. Luciferase Reporter Assay

Lucia reporter plasmid pCpGfree-basic-Lucia was purchased from InvivoGen (San Diego, CA, USA); the plasmid had no promoter and was devoid of CpG dinucleotides. The reporter vector pCpGfree-MT–264/+10 was constructed as in [18]; the vector contained the mouse MT1 promoter (bases −264 to +10 relative to the transcription start site). Point mutants and deletion mutants were constructed by inverse PCR using the PrimeSTAR Mutagenesis Basal Kit (Takara Bio) or by cloning at HindIII/NcoI sites, with synthetic double-stranded DNA fragments (Integrated DNA Technologies, Coralville, IA, USA) (Supplementary Table S1). Firefly luciferase reporter control plasmid pGL4.12-SV40 was constructed using pGL4.12 [*luc2CP*] (Promega, Madison, WI, USA), SV40 enhancer, and the early promoter fragment obtained from pRL-SV40 (Promega) and In-Fusion Cloning Kit (Takara Bio). Lucia reporter vectors were methylated with CpG methyltransferase (M.SssI; CG→^5m^CG; New England Biolabs, Ipswich, MA, USA) or HhaI methyltransferase (GCGC→G^5m^CGC; New England Biolabs) for 1 h at 37 °C (Figure 2). Methylated reporter vectors were purified through extraction with NucleoSpin Gel and PCR Clean-up (TaKaRa Bio). Methylation completeness was confirmed using the methylation-sensitive HhaI restriction enzyme.

Transfection was performed with transfection reagent FuGENE HD (Promega, Madison, WI, USA), according to the manufacturer’s protocol. In brief, the reporter vector, pGL4.12-SV40 and pEGFP were mixed at a 1:0.005:1 ratio in Opti-MEM medium (Invitrogen, Carlsbad, CA, USA). The plasmid mixture (0.3 μg per well) was mixed with FuGENE HD (2.0 μL per well), incubated for 15 min at room temperature, added to the cells cultured in a 24-well plate, and cultured for 24 h. The cells were lysed in 1× passive lysis buffer (Promega), and luciferase activity was measured using the Dual-Luciferase Reporter Assay System (Promega) and GloMax 20/20n luminometer (Promega).

### 3.6. Statistical Analysis

Data were analyzed with Tukey’s test in PASW Statistics 18 software (IBM, Armonk, NY, US). Differences between groups were considered significant at *p* < 0.05.

## 4. Conclusions

In the present study, we found that methylation/demethylation of the CpG sites near MREd and MREe is important for regulation of *Mt1* gene expression. Our findings suggest that *Mt1* gene expression is fine-tuned through epigenetic regulation. To understand the exact regulation mechanisms of *Mt1* gene expression, it is necessary to clarify which CpG sites or which CpG regions are important, more definitely. Site-specific methylation/demethylation techniques [29,30] are needed to clarify the important sites and regions. On the other hand, our finding was from the experiments using exogenous *Mt1* gene promoter-driven Lucia luciferase reporter vectors. It is important to clarify whether ununiformed methylation/demethylation of the CpG sites also regulates endogenous *Mt1* gene expression and what environmental factors influence CpG methylation and modify *Mt1* gene expression. As MT1 plays a central role in defense against heavy metals and ROS, the fine-tuned *Mt1* gene expression might affect many biological functions. Further investigation is needed to explore the biological role of the non-uniform role of specific CpG sites in the regulation of MT1 expression.

## Figures and Tables

**Table 1:** Relative metallothionein-1 (MT1) and β-actin mRNA levels after 5-AzaC and Cd treatment.

Gene	Control	Cd	5-AzaC + Cd
MT1	1.0 ± 1.9	0.9 ± 0.7	31,214.4 ± 16,310.7 *
β-actin	1.0 ± 0.6	0.9 ± 0.3	0.9 ± 0.5

Expression levels were normalized using 18S rRNA. Values are expressed as the means ± SD from 3–4 independent experiments. * *p* < 0.01 versus the control group.

## Supplementary Materials

The following is available online at https://www.mdpi.com/1422-0067/21/17/5946/s1, Table S1: Primer and synthetic DNA sequences for construction of reporter vectors.

## Abbreviations

MT	Metallothionein
ROS	Reactive oxygen species
MRE	Metal response element
MTF1	MRE-binding transcription factor-1
HAT	histone acetyltransferase
5-AzaC	5-azacytidine
MEF	Mouse embryonic fibroblast
MTF1-KO	MTF1-knockout
qPCR	quantitative PCR
