# Supplementary material for: CpG Site-Specific Regulation of Metallothionein-1 Gene Expression
Source: Int J Mol Sci. 2020 Aug 19;21(17):5946. doi: 10.3390/ijms21175946 (PMC7503544; doi:10.3390/ijms21175946)
Supplement: Supplementary file 1 [file ijms-21-05946-s001.pdf]

Supplementary Table S1 Primer and synthetic DNA sequences for construction of reporter vectors.

	Target	Template		Sequence
Inverse PCR	Δ–11/+42	pCpGf-WT	F	5′-CCGCCCGAGACTATAAAGAGGGCAGG-3′
			R	5′-TATAGTCTCGGGCGGAGTGCAGAGC-3′
	ΔMREabc	pCpGf-WT	F	5′-AAGCTTGAAAAGTGCGCTCGGCTCTG-3′
			R	5′-GCACTTTTCAAGCTTTGCATGCTAC-3′
	ΔMREde	pCpGf-WT	F	5′-CAAAGCTTTGCACACTGGCGCTCCAG-3′
			R	5′-GTGTGCAAAGCTTTGCATGCTACTA-3′
	m14 CG→CC	pCpGf-m15-21	F	5′-GCACTCAGCCCGAAAAGTGCGCTCGG-3′
			R	5′-TTTCGGGCTGAGTGCAGAGCTCCCTG-3′
	m15 CG→CC	pCpGf-WT	F	5′-CACTGGCCCTCCAGGGAGCTCTGCAC-3′
			R	5′-CCTGGAGGGCCAGTGTGCACAGCGGG-3′
	m8 CG→CC	pCpGf-WT	F	5′-GCGGGGCCCGTGACTATGCGTGGGCT-3′
			R	5′-GTCACGGGCCCCGCGTCCTTGGCAG-3′
Synthetic DNA fragment	m15-21			5′-TGGCCTCGGCGGCCAAGCTTGATAGGCCGTAATATCGGGGAAAGCACTAT<br>AGGGACATGATGTTCCACACCTCACATGGGTCCTCCTATCCCAGCCAGTCCT<br>GCCAAAGGGGGGGTCCCCCTGTGCACACTGGCCCTCCAGGGAGCTCTGCACT<br>CCGCCCGAAAAGTGCGCTCGGCTCTGCCAAGGACGCGGGGCGCGTGACTATG<br>CGTGGGCTGGAGCAACCGCCTGCTGGGTGCAAACCCTTTGCGCCCGGACTCG<br>TCCAACGACTATAAAGAGGGCAGGCTGTCCTCTAAGCGTCACCACGACTTCA<br>ACGTCCTGAGTACCTTCCCATGGAAGATGCCAAAAACATT-3′
	m1-12			5′-TGGCCTCGGCGGCCAAGCTTGATAGGCCGTAATATCGGGGAAAGCACTAT<br>AGGGACATGATGTTCCACACGTCACATGGGTCGTCCTATCCGAGCCAGTCGT<br>GCCAAAGGGGCGGTCCCGCTGTGCACACTGGCGCTCCAGGGAGCTCTGCACT<br>CCGCCCGAAAAGTGCACTCAGCTCTGCCAAGGAGGGGGGGCCCCTGACTATG<br>CCTGGGCTGGAGCAACCCCCTGCTGGGTGCAAACCCTTTGCACCCAGACTCC<br>TCCAACCACTATAAAGAGGGCAGGCTGTCCTCTAAGCCATGGAAGATGCCAA<br>AAACATT-3′

F: forward primer, R: reverse primer. To construct pCpGf-m14-21, CpG14 in pCpGf-m15-21 was mutated by inverse PCR. Mutated CpG dinucleotides are underlined.
